# Treatment of Snoring with a Nasopharyngeal Airway Tube

**DOI:** 10.1155/2016/3628716

**Published:** 2016-10-04

**Authors:** Macario Camacho, Edward T. Chang, Camilo Fernandez-Salvador, Robson Capasso

**Affiliations:** ^1^Otolaryngology-Head and Neck Surgery, Division of Sleep Surgery and Medicine, Tripler Army Medical Center, 1 Jarrett White Road, Honolulu, HI 96859, USA; ^2^Department of Psychiatry and Behavioral Sciences, Division of Sleep Medicine, Stanford Hospital and Clinics, Redwood City, CA 94063, USA; ^3^Department of Otolaryngology-Head and Neck Surgery, Division of Sleep Surgery, Stanford Hospital and Clinics, Stanford, CA 95304, USA

## Abstract

*Objective*. To study the feasibility of a standard nasopharyngeal airway tube (NPAT) as treatment for snoring.* Methods*. An obese 35-year-old man, who is a chronic, heroic snorer, used NPATs while (1) the patient's bedpartner scored the snoring and (2) the patient recorded himself with the smartphone snoring app “Quit Snoring.” Baseline snoring was 8–10/10 (10 = snoring that could be heard through a closed door and interrupted the bedpartner's sleep to the point where they would sometimes have to sleep separately) and 60–200 snores/hr. Several standard NPATs were tested, consisting of soft polyvinyl chloride material raging between 24- and 36-French (Fr) tubes.* Results*. The 24 Fr tube did not abate snoring. The 26 Fr tube was able to abate the snoring sound most of the night (smartphone app: 11.4 snores/hr, bedpartner VAS = 2/10). The 28 and 30 Fr tubes abated the snoring sound the entire time worn (smartphone app: 0 snores, bedpartner VAS 0/10) but could not be tolerated more than 2.5 hours. The tube of 36 Fr size could not be inserted, despite several attempts bilaterally.* Conclusion*. Appropriately sized nasopharyngeal airway tubes may abate the snoring sound; however, as in this patient, they may be too painful and intolerable for daily use.

## 1. Introduction

Snoring is a common problem throughout the United States and worldwide, with literature reporting that 5–86% of men and 2–47% of women snore [1, 2]. The simple act of changing from an upright to a supine position has been known to decrease the size of the upper airway by approximately one-third in patients with obstructive sleep apnea (OSA) [3]. Therefore, lying supinely likely can narrow the upper airway because of gravity, even in patients without OSA, which can predispose patients to snoring. Usually snoring is due to the palate; however, snoring can occasionally be due to the tongue or even the epiglottis (in approximately 12% of patients) [4]. Several medical and surgical modalities have been evaluated to treat snoring; however, to our knowledge none has reported the use of a standard nasopharyngeal airway tube [5–7]. Polyvinyl chloride nasopharyngeal airway tubes (NPATs) are readily available and are commonly used by anesthesiologists for patients either during induction or in the immediate postoperative period to help prevent obstruction of the airway [8–10]. NPATs have also been used to treat obstructive sleep apnea with mixed results [11, 12]. The objective of this study was to evaluate the feasibility of a standard nasopharyngeal airway tube as treatment for snoring.

## 2. Methods

MEDLINE, Scopus, and the Cochrane Library were searched from inception to April 8, 2016, for treatment of snoring with a nasopharyngeal airway tube. Terms searched included the various combinations of snoring and nasal or nasopharyngeal and trumpet, airway, obturator, or tube. There were no studies describing the use for snoring treatment. The Stanford IRB was then contacted, and written approval for this case study was obtained prior to initiating this study.

The patient is a 35-year-old man, who is a chronic snorer with a BMI of 32.4 kg/m^2^ who is not sleepy (Epworth Sleepiness Scale = 1/24). The patient's wife is a very light sleeper and is highly sensitive to snoring sounds. Over the past 14 years, the patient's weight has gone as high as 129.5 kg (BMI = 39.7 kg/m^2^) and as low as 88.2 kg (BMI = 27.1 kg/m^2^). The patient is known to have heroic snoring confirmed by his spouse for several years, when his BMI is above 100 kg (BMI = 30.7 kg/m^2^); however, at weights below 100 kg, there is no snoring noted. The patient's current weight has been relatively unchanged (±1 kg) for the past year. History and physical exam demonstrate that the patient has no nasal septal deviation [13] and no inferior turbinate hypertrophy (grade 2 bilaterally [14]) and has not had any complaints of nasal obstruction (NOSE questionnaire score = 10/100). After reviewing the literature for the variety of published modalities to abate snoring, the patient selected to utilize a previously unpublished technique for snoring, which is the use of a standard nasopharyngeal airway tube. This modality was selected since the patient has no nasal obstruction, septal deviation, or turbinate hypertrophy; therefore, the anatomy should be conducive to placement and use of NPAT. The sizes of soft polyvinyl chloride nasopharyngeal airway tubes evaluated included 24, 26, 28, 30, and 36 French (Fr).

To objectively monitor the results of the snoring sound, the patient used a smartphone app. A recently published study evaluated smartphone snoring apps which were rated by 4 authors who downloaded the apps on iTunes and scored and ranked them [15]. The snoring app “Quit Snoring” was the highest rated app amongst all the iTunes apps [15]. It was compared to polysomnography as part of the same study and was noted to have a high positive predictive value between 93 and 96%; therefore, the smartphone app was used for this study. Quit Snoring [15] was developed by Pointer Software Systems, Ltd., and could record the snoring sound and allowed for graphic visualization of the snoring sound to facilitate playback. The smartphone was set up in bed with the microphone facing towards the patient. The phone was calibrated to ensure that any snoring sounds would be accurately identified. The recordings were then reviewed the next morning, and any noises or sounds created that were above the baseline environmental noises were individually evaluated to determine if the sound was a snoring sound, patient movement, or environmental noise. A log was kept for time and descriptions for snoring sounds, pain, discomfort, and symptoms during and after the insertions of the NPATs. In order to quantify the effect of NPATs on snoring, the patient used NPATs while (1) the patient's bedpartner scored the snoring each morning after the NPATs were used and (2) the patient recorded himself with the smartphone snoring app “Quit Snoring.” Baseline snoring was 8–10/10 (10 = snoring that could be heard through a closed door and interrupted the bedpartner's sleep to the point where they would sometimes have to sleep separately) and 60–200 snores/hr.

## 3. Results

### 3.1. First NPAT Test Night

A NPAT of 36 Fr size could not be inserted; however, a NPAT of 30 Fr size was successfully positioned into the nasal cavity with extension into the nasopharynx and oropharynx. The NPAT's extension into the oropharynx was confirmed visually and with transoral and neck palpation. The patient attempted to produce a snoring sound, but it was not possible. There was great difficulty in swallowing as well. By midnight, the patient became desensitized to the NPAT and went to bed. The patient went to sleep and then, at 2:12 am, awoke with a nightmare and significant (8/10) right nasal cavity pain and he quickly removed the NPAT from the nares. The snoring index was 0 events per hour and the bedpartner visual analog scale score was 0/10. The patient attempted to reinsert the tube for 30 minutes and could not, so the patient went back to bed at 2:44 am. The residual pain from the large diameter and repeated insertion attempts made it difficult to go back to sleep; however, by 3:49 am, the patient was snoring heroically again and continued to snore (259 events) until waking at 6:20 am.

Initially, the patient had planned to use the nasopharyngeal airway on consecutive days; however, the patient developed significant nasal obstruction, facial pressure, and a constant, dull headache. However, after 2 days the symptoms subsided, so the patient then continued to evaluate the NPATs.

### 3.2. Second NPAT Test Night

A tube of 30 Fr size was inserted into both nares, and, despite being difficult to pass, it was able to be kept in place for about 10 minutes. The NPAT was removed secondary to pain. The tube of 28 Fr size was placed successfully on the right side and after 20 minutes the patient desensitized himself to the tube and went to bed at 12:23 am, slept with the NPAT in place until 1:09 am, and then removed it after waking up secondary to pain (7/10) and discomfort. The snoring index was 0 events per hour and the bedpartner visual analog scale score was 0/10. The next morning, patient developed similar symptoms to the previous time he used the NPAT and had a dull headache, tooth pain, and retroorbital pain on the right and nasal obstruction. After two days, the symptoms subsided enough to proceed with further evaluation of the NPATs.

### 3.3. Third NPAT Test Night

**Figure 1:**
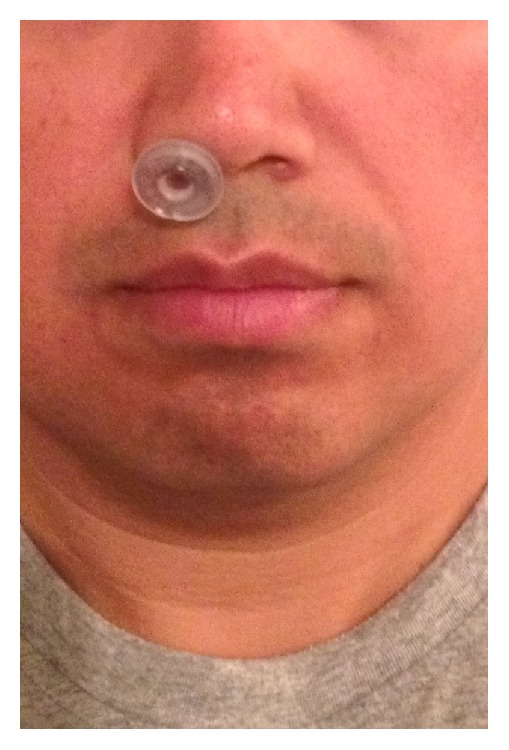
Size 26 nasopharyngeal airway tube in place prior to sleep.

Tubes of 30 Fr and 28 Fr size were inserted but were not tolerated secondary to pain which transmitted to the right maxillary sinus and right retroorbital region. A tube of 24 Fr size was placed easily; however, with every swallow the NPAT would extrude and, even with the NPAT fully positioned, the patient could produce a snoring sound. The patient then decided to try the tube of 26 Fr size, which proved to be comfortable, and a snoring sound could not be produced, and it remained in position with swallowing (Figure 1). The patient went to bed at midnight and produced one snoring sound at 1:07 am (55 dB), one at 2:43 am (55 dB), and twenty snoring sounds at 2:54 am (55–60 dB) for a rate of 11.4 snores per hour. The bedpartner's visual analog scale score was 2/10. The patient then awoke at 4:11 am, had moderate nasal (4/10) and throat discomfort, and removed the tube. The patient then went back to bed and had difficulty falling asleep initially but eventually fell asleep and started snoring at 5:24 am (50 episodes above 50 dB each) and subsequently woke up to get ready for work at 5:38 am.  Table 1 summarizes the 3 nights that the NPATs were used.

## 4. Discussion

Although anesthesiologists have used soft polyvinyl chloride nasopharyngeal airway tubes with great success in the pharmacologically induced sleep or anesthetized state for preventing airway obstruction, the utility of these tubes for snoring abatement is met with challenges. The NPAT sizes that abated the snoring sound completely in this patient were tubes of 28 and 30 Fr sizes. These two sizes, however, could not be tolerated more than 2.5 hours during the night secondary to pain, despite the patient having desensitized himself to having the tube in place while awake between 20 and 30 minutes before going to sleep. After the initial pain of insertion, there was no significant amount of pain until awakening during sleep, during which time there was a significant amount of pain (7-8/10). The tube of 26 Fr size was able to prevent the snoring sound; however, like tubes of 28 and 30 Fr size, it produced discomfort enough to warrant removal. Although the tube of 26 Fr size did allow for snoring while patient was asleep, this may have been due to the tube partially sliding out of the nares. There is no way to know this since the patient was asleep when it happened and did not notice it to have been partially out upon awakening. The use of a head strap could have ensured that the NPAT was not sliding out during sleep; however, the patient did not want to put pressure on the nares during sleep.

Deterrents to using NPATs in this patient included headache, discomfort, pain, and nasal obstruction. It is possible that a turbinoplasty would have made the NPATs' use tolerable; however, since the patient had no nasal obstruction at baseline, he did not want to pursue a turbinoplasty simply as a means to wear the NPATs during sleep. There are potential problems for snoring patients who have a deviated nasal septum or have large inferior turbinates; the side effects include (1) headache, (2) epistaxis, (3) discomfort, (4) pain, (5) rebound nasal obstruction after removal in the daytime, (6) synechiae (scar tissue with adhesion) if there are excoriations between the nasal septum and turbinates, and (7) saliva/mucous draining from the NPAT during sleep.

If future researchers are going to evaluate standard NPATs for snoring treatment then the patient's nasal anatomy is crucial to the success and failure. In this study subject, although there was no septal deviation or turbinate hypertrophy, the patient likely developed edema over the period of inserting and leaving the NPATs in position for the hours that it was used. Despite not having turbinate hypertrophy and being able to place the NPATs and have desensitization, the 4-hour nasal cycling of the turbinates likely created edema on the side of use, which may have contributed to the significant pain and awakening during sleep. Although it is possible that the NPATs affected sleep, the patient was able to dream while they were in place, so if there were to be snoring, it should have occurred since the patient is a heroic snorer as demonstrated by resumption of the snoring sound after removal of the NPATs. It is possible that NPATs would be more tolerable if the patients undergo bilateral inferior turbinoplasties. Additionally, another type of nasopharyngeal airway tube can be tried by chronic snorers, which is the AlaxoStent [16], which consists of a smooth tubular shape memory metal (braided) and may be more tolerable for patients.

## 5. Conclusion

The appropriately sized nasopharyngeal airway tube for patients may abate the snoring sound; however, as in this patient, it may be intolerable for daily use.

## Figures and Tables

**Table 1:** Soft polyvinyl chloride nasopharyngeal airway tube sizes and insertion results.

NPAT^*∗*^ sizes (Fr)	Length	Outer diameter	Ability to be inserted	Hours tolerated during sleep	Outcomes from insertion and use during sleep
36	180 mm	12.0 mm	N	NA	Tube kept hitting the inferior turbinate and could not pass.

30	165 mm	10.0 mm	Y	2.5	Tolerated insertion for 2.5 hours and removed tube due to pain. Smartphone app: 0 snores. Bedpartner: 0/10 VAS.

28	155 mm	9.3 mm	Y	0.75	Tolerated insertion for 0.75 hours and removed tube due to pain. Smartphone app: 0 snores. Bedpartner: 0/10 VAS.

26	140 mm	8.7 mm	Y	4.2	Tolerated insertion 4.2 hours and snored during sleep. Smartphone app: 11.4 snores per hour. Bedpartner: 2/10 VAS.

24	130 mm	8.0 mm	Y	NA	Tube was easily inserted, however, was too short to stop simulated snoring, and therefore was not used overnight.

(Note: manufacturer is Teleflex; dimensions are also available at http://www.teleflex.com/.) Fr = French; ^*∗*^NPAT = nasopharyngeal airway tube; NA = not applicable; and VAS = visual analog scale (0 = no snoring, 10 = snoring that could be heard through a closed door and significantly interrupted the bedpartner's sleep).
